# Mr. Hume's Observations on Dr. Roget's Reply

**Published:** 1813-01

**Authors:** Joseph Hume

**Affiliations:** Long Acre, London


*To the Editors of the Medical and Physical Journal.*

GENTLEMEN,

DR. ROGET having now confessed that I have made use of the words "or any other alkali," the point at issue between us becomes more defined, and the question reduced to a very simple form, namely,—Whence are those distinctions between *sulphate of copper* and *nitrate of silver*, which prove the one to be *totally* different from the other, as a test for arsenic?

That there is a total difference, especially as far as ammonia is concerned, has been asserted by Dr. Roget:—that there is *no* such distinction between the application of these tests to warrant such an expression, is what I shall now endeavor to prove.

Had it not been for this assertion, I should not have occasion to address you again on this subject; since Dr. Roget has, I think with great propriety, dropped all claim in favor of Dr. Marcet as the discoverer of a *test* for arsenic, and reduced, what he is pleased to call, "the controversy" to "the *priority* of the application of ammonia."

Let us now compare the two tests, and see if there be any other essential difference than that which was originally noticed by myself in the Philosophical Magazine, and in these words: "The test which I propose as a substitute (to sulphate of copper) appears to be more efficacious, in as much as it produces *a more copious precipitate* from a *given quantity* of arsenic."

I shall here draw a parallel of comparison, by stating, in the first place, that each of these salts has a metallic base, and both serve as tests for the same purpose, admitting of variations precisely similar in respect to their *application*.

2. The addition of an *alkali* to the arsenic, is as necessary for one of these salts as for the other; and as much caution is required that this be not in excess.

3. Any of the alkaline *earths* may be taken for the process, whether the chief agent be *copper* or *silver*. I may just hint, in addition to what I have lately published in the Philos. Magaz.* that sulphate of copper is likely to produce a more dense and obvious effect than nitrate of silver, when native carbonate of *barytes* has been chosen as the alkaline medium.

4. Both the copper and silver tests act exactly upon the same chemical principles, or, in the language of Bergman, by double elective attraction.

* October, 1812.

5. It

5. It is altogether in my favor that these metallic salts have a capacity of forming ternary and permanent compounds with *ammonia.* Of this fact Dr. Marcet and Dr. Roget seem totally ignorant, otherwise they would not have applied the two fluids *separately,* and in such a vague manner; when, by uniting them, agreeably to the method I have prescribed, a more perfect test might have been obtained. Dr. Roget need not wonder, therefore, that I should become the "panegyrist" of what is exclusively my own invention, that of combining the alkali with the *test* itself, instead of with the arsenic. Here there is a striking resemblance in the two tests; for, as we can readily form the triple compound of *ammoniaco-nitrate of silver,* we can have also an ammoniaco-sulphate, nitrate, &c. of *copper.* I trust I need go no further in search of arguments to shew, that, whatever these salts may be in other respects, they are not *totally* different from each other as tests for arsenic; but, on the contrary, are identical in their application, conformably to the most approved chemical theory.

6. Both these tests are remarkable for producing *abundant* precipitates, and hence they exceed all other arsenical tests. The silver is, however, as I first noticed, by far the most delicate and conspicuous, and, consequently, the most efficacious.

Lastly. There is a close analogy between the copper and silver in their agency upon arsenic; either of them will distinguish, by the *color* of the precipitate, in what state the poison exists in respect to its oxygen. This property makes them valuable to the analyst; and it likewise goes far to establish that similarity which has been so positively denied to exist.

So little, indeed, do these tests differ in their application, that I might have announced the silver-test I originally proposed, by a single line, and not a whole page, for my first letter did not occupy more. I might have said, "Let nitrate of silver be substituted for sulphate of copper, and you will have a more powerful test for arsenic;" and then left all scrambling and quibbling about alkalies to be disputed by others.

It is evident, that the particular species of alkali to be used, was a mere secondary consideration with me, in the experiments with which I commenced the inquiry; for, in the letter already quoted, I have said, "though my process answers very well with potass, or even lime-water, yet I am inclined to prefer the common subcarbonate of soda."*

* Philos. Mag. May, 1809.

Upon

Upon the whole, it appears that the words "or any other alkali" have served as a palpable hint, of which every advantage has been taken. Dr. Roget's paper, submitted to the Medical and Chirurgical Society, I believe to be highly valuable; the chemical matters are, however, somewhat exceptionable, independently of that assumption of "*another test which occurred to Dr. Marcet*," and which, says Dr. Roget, "I am happy in having this opportunity of communicating to the Society." In the course of this discussion, I have pointed out and proved the existence of some errors, but to comment on others, not immediately concerned with my claim, is what I cannot encourage; therefore, lest I appear vindictive, I shall pass them by, and proceed to the remainder of this letter, which, I sincerely hope, will be the last upon this subject,—the vindication of my prior claim to the arsenical test by means of *silver*.

In quoting Dr. Bostock, Dr. Roget has named sulphate of copper only—not a word about the *alkali;* and, had Dr. Roget done me equal justice, he would have given my test also its proper name, that of *nitrate of silver*, which is in fact its proper title. Dr. Marcet's fame, as a philosopher and a chemist, is very firmly established: it wanted no support at my expence, especially when Dr. Roget must be conscious of my priority in point of time; and that, in respect to the powers which the test possesses, the important agent is the silver, which can act *without* ammonia, while this alkali is of no use without silver or copper, as I have just shewn in this communication. I remain, Gentlemen,

*Long Acre, London,* Your obedient Servant,

*Dec.* 14, 1812. JOSEPH HUME.

